# Editorial: Insights in aquatic microbiology: 2022

**DOI:** 10.3389/fmicb.2023.1250444

**Published:** 2023-07-19

**Authors:** Jin Zhou, Susana Agusti

**Affiliations:** ^1^Shenzhen Public Platform for Screening and Application of Marine Microbial Resources, Institute for Ocean Engineering, Shenzhen International Graduate School, Tsinghua University, Shenzhen, China; ^2^Red Sea Research Center (RSRC), King Abdullah University of Science and Technology (KAUST), Thuwal, Saudi Arabia

**Keywords:** microorganisms, marine, freshwater, aquatic microbiology, editorial

Aquatic microbiology is a diverse and expansive field that focuses on studying the interactions and dynamics of microorganisms in various aquatic ecosystems, including oceans, lakes, rivers, and other water bodies. In recent years, there have been significant advancements and discoveries in this field, contributing to our understanding of the crucial role microorganisms play in aquatic ecosystems. The journal section Aquatic Microbiology, which is part of the journal Frontiers in Microbiology and Frontiers in Marine Science, is a notable one that has emerged as a leading publication in the topic of aquatic microbiology (Figure 1). The search on the Web of Science of “aquatic” (all fields) AND “microbiology” (all fields) for the year 2022 yielded 372 publications, with a significant proportion (32.8%) published in this particular section (Figure 1A). These publications covered several of the Web of Science categories contributing moreover to general microbiology, to biotechnology and applied microbiology, and environmental sciences among, others (Figure 1B). Aquatic microorganisms are a focal point of research, particularly in areas such as biogeochemical cycling, material metabolism characteristics, pollutant degradation, ecological restoration, and public health. Understanding the role and behavior of aquatic microorganisms in these contexts is crucial for advancing scientific knowledge and addressing environmental challenges.

**Figure 1:**
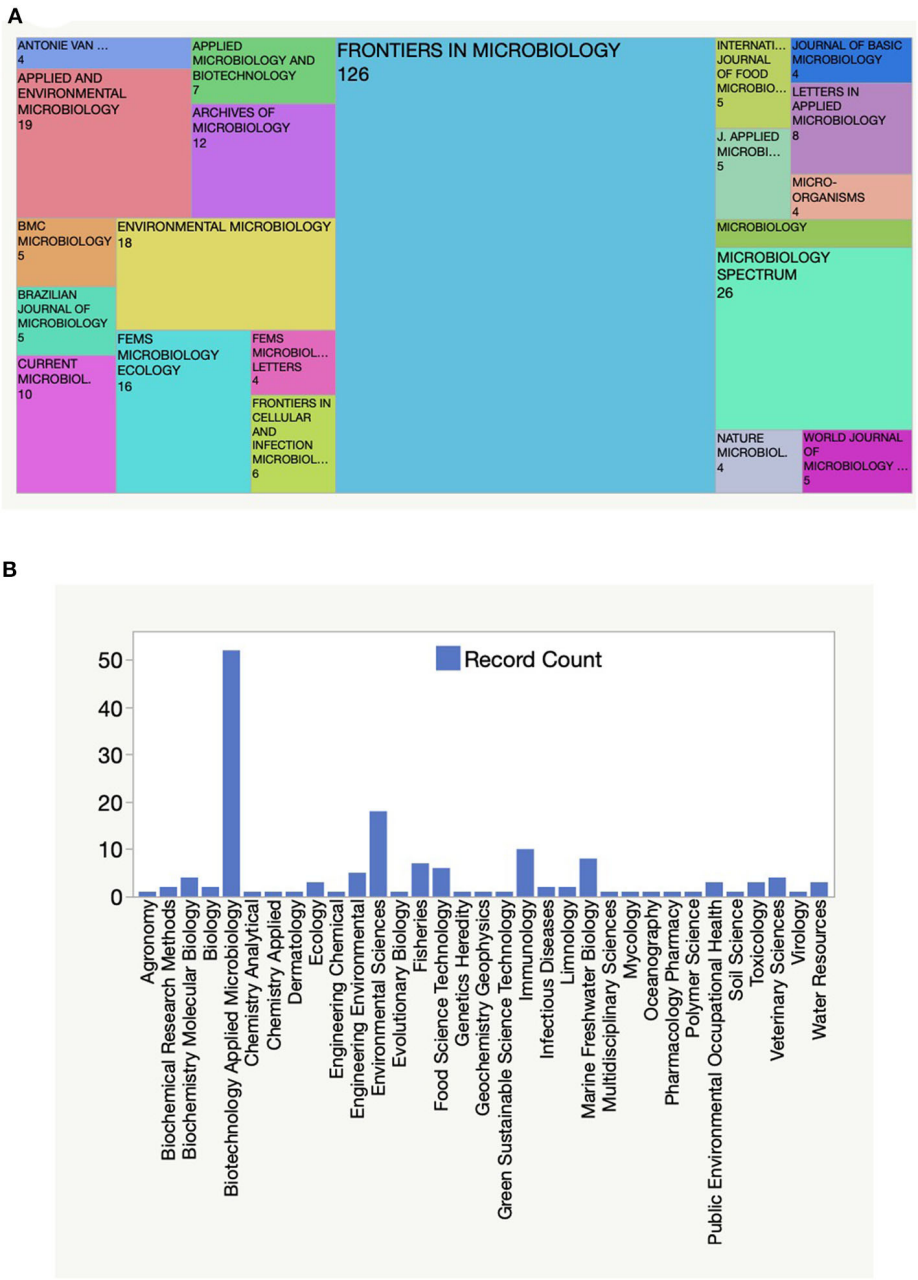
Titles and corresponding number of publications in journals that publish papers on the topic of Aquatic Microbiology during 2022 **(A)**. Information on the number of publications categorized under different topics within the search for Aquatic Microbiology **(B)**. These data have been obtained from the Web of Science.

In recent years, there have been several new insights and discoveries in this field. The publications included in the present Research Topic “*Insights in aquatic microbiology: 2022”* encompass a range of topics that further expand our knowledge in this field, addressing the following aspects:

1. Pan et al. [*Transcriptome, intestinal microbiome, and histomorphology profiling of differences in the response of Chinese sea bass (Lateolabrax maculatus) to Aeromonas hydrophila infection*] investigates the genetic and microbial factors that influence the response of Chinese sea bass to infection by *Aeromonas hydrophila*. It provides a reference for the study of the immune response and particular functions of intestinal microbes of sea bass after pathogen infection.
2. (*Elevated CO*_2_ *reduces copper accumulation and toxicity in the diatom Thalassiosira pseudonana*) the study of Xu et al. explores the impact of increased carbon dioxide (CO_2_) levels on copper accumulation and toxicity in the diatom *Thalassiosira pseudonana*, highlighting the potential effects of environmental changes on microorganisms. This work provides a basis for a better understanding of the bioremediation capacity of marine algae, which may have profound effect on the security of seafood quality and marine ecosystem sustainability under further climate change.
3. Zhai et al. (*Ocean acidification alters the benthic biofilm communities in intertidal soft sediments*) focuses on the effects of ocean acidification on the composition of benthic biofilm communities on intertidal soft sediments, shedding light on the ecological consequences of changing ocean chemistry. This study suggests that benthic biofilms in intertidal sandy sediments are likely to change significantly near the end of the century if anthropogenic CO_2_ emissions unmitigated, with profound implications on local ecosystems and biogeochemical cycling.
4. Zhu et al. (*Microbial community composition and metabolic potential during a succession of algal blooms from Skeletonema sp. to Phaeocystis sp*.) investigated the changes in microbial community composition and metabolic potential during the transition from algal blooms dominated by *Skeletonema* sp. to those dominated by *Phaeocystis* sp., providing insights into the complex interactions between microorganisms and algal blooms. The phycosphere microbial behavior (community and function) might be an internal driving factor for the algal bloom succession.
5. Stojan et al. (*Evaluation of DNA extraction methods and direct PCR in metabarcoding of mock and marine bacterial communities*) assessed different DNA extraction methods and the use of direct PCR in metabarcoding analysis of mock and marine bacterial communities. The research aims to improve the accuracy and efficiency of studying microbial communities in marine environments. It can help us making a cautious decision about the choice of the extraction method or direct PCR approach.
6. Ren et al. (*Isolation and characterization of algicidal bacteria from freshwater aquatic environments in China*) focused on the isolation and characterization of algicidal bacteria from freshwater aquatic environments in China, which contributes to our understanding of the ecological roles of these bacteria. This work supplied us a new method to using algicidal bacteria inhibit the harmful algal blooms.

These publications, among others, contribute to the advancement of aquatic microbiology by providing new insights into the ecology, interactions, and responses of microorganisms in aquatic ecosystems. They help shape our understanding of the intricate microbial dynamics and the importance of microorganisms in maintaining the health and functioning of aquatic environments. It should be pointed out that the research on aquatic microorganisms is currently developing rapidly. In order to better discover and solve scientific problems, on the one hand, we need to develop more novel technologies, such as artificial intelligence, machine learning, etc., to help us to analysis the big data; on the other hand, we also need innovative scientific thinking, focusing on and solving real problems, breaking away from the current tracking research, so that microbial research can better serve the ecological environment and global biosphere. This Research Topic provides us with a window and some hope, and we believe that future microbial research will be bright.

## Author contributions

All authors listed have made a substantial, direct, and intellectual contribution to the work and approved it for publication.

